# Interfacially Induced Cascading Failure in Graphite‐Silicon Composite Anodes

**DOI:** 10.1002/advs.201801007

**Published:** 2018-12-14

**Authors:** Seoung‐Bum Son, Lei Cao, Taeho Yoon, Arthur Cresce, Simon E. Hafner, Jun Liu, Markus Groner, Kang Xu, Chunmei Ban

**Affiliations:** ^1^ National Renewable Energy Laboratory 15013 Denver West Parkway Golden CO 80401 USA; ^2^ Department of Material Science and Engineering Massachusetts Institute of Technology Cambridge MA 02139 USA; ^3^ School of Chemical Engineering Yeungnam University Gyeongsan 38541 Republic of Korea; ^4^ Electrochemistry Branch Sensor and Electron Devices Directorate U.S. Army Research Laboratory Adelphi MD 20783‐1197 USA; ^5^ Department of Mechanical Engineering University of Colorado 596 UCB Boulder CO 80309 USA; ^6^ ALD NanoSolutions 580 Burbank Street, Unit 100 Broomfield CO 80020 USA

**Keywords:** energy storage, lithium‐ion batteries, molecular layer deposition, silicon anodes, solid electrolyte interphase

## Abstract

Silicon (Si) has been well recognized as a promising candidate to replace graphite because of its earth abundance and high‐capacity storage, but its large volume changes upon lithiation/delithiation and the consequential material fracturing, loss of electrical contact, and over‐consumption of the electrolyte prevent its full application. As a countermeasure for rapid capacity decay, a composite electrode of graphite and Si has been adopted by accommodating Si nanoparticles in a graphite matrix. Such an approach, which involves two materials that interact electrochemically with lithium in the electrode, necessitates an analytical methodology to determine the individual electrochemical behavior of each active material. In this work, a methodology comprising differential plots and integral calculus is established to analyze the complicated interplay among the two active batteries and investigate the failure mechanism underlying capacity fade in the blend electrode. To address performance deficiencies identified by this methodology, an aluminum alkoxide (alucone) surface‐modification strategy is demonstrated to stabilize the structure and electrochemical performance of the graphite‐Si composite electrode. The integrated approach established in this work is of great importance to the design and diagnostics of a multi‐component composite electrode, which is expected to be high interest to other next‐generation battery system.

## 1 Introduction

Developing sustainable, inexpensive, and high‐energy‐density Li‐ion batteries (LIBs) is vital to realize electrified transportation and deepen the penetration of renewable energy.^1^ As part of the comprehensive efforts, silicon (Si) has been considered as a potential candidate to replace the current anode material, graphite (372 mAh g^−1^, LiC_6_), because of its high capacity (3579 mAh g^−1^, Li_15_Si_4_),^2^, ^3^, ^4^ low environmental impacts, and low cost. Noteworthy advances have been made in addressing Si's instabilities through the design of nanostructured Si,^5^, ^6^, ^7^, ^8^, ^9^, ^10^, ^11^ functional conductive polymer binders^12^, ^13^, ^14^, ^15^ for mitigating the mechanical degradation of Si, and electrolyte additives^16^, ^17^, ^18^ to form a more stable solid electrolyte interphase (SEI). However, the volume expansion of Si anode material, a consequence of the high lithiation degree of Si, continues to cause poor cycling stability associated with mechanical degradation and hinders the use of high‐capacity Si in commercial rechargeable LIBs.^3^, ^19^, ^20^, ^21^, ^22^

To alleviate mechanical instabilities and simultaneously improve energy density of Si anodes, composite electrodes comprising graphite and Si (G–Si) at various ratios have increasingly been investigated.^23^, ^24^, ^25^, ^26^ Such an approach, which incorporates a small portion of Si into the graphite‐matrix, was proposed to achieve a higher energy density compared to the graphite‐only anode, but with less volumetric change as compared to the Si‐only anode. However, G–Si composite electrodes still suffer from capacity fade and insufficient Coulombic efficiency (CE). In this research, we provide a calculus‐based method using differential plots and integrations to differentiate the individual electrochemical behaviors of the graphite and Si components in such composite, and to identify the failure mechanism therein. A surface modification enabled by a molecular layer deposition (MLD) technique, recently demonstrated in our research group,^27^, ^28^, ^29^, ^30^, ^31^, ^32^, ^33^ has been applied on the G–Si composite electrodes to stabilize the surface of the Si component. With this artificial interphase layer, we demonstrate highly reversible G–Si electrodes with a specific capacity of ≈810 mAh g^−1^ (2 mAh cm^−2^) for hundreds of charge–discharge cycles.

## 2 Results and Discussion

### 2.1 Failure Analysis of the G–Si Electrodes Using Calculus Methods

**Figure 1:**
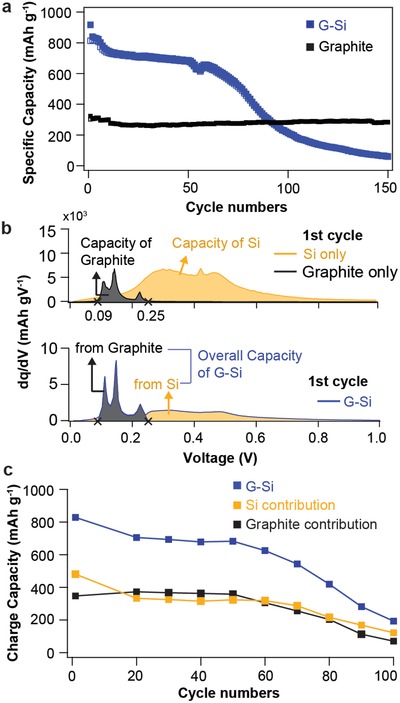
Electrochemical performances of the G–Si composite electrode. a) Cycling stability of G–Si and graphite electrode. b) First‐cycle d*Q*/d*V* for G–Si, Si, and graphite electrode. c) Charge‐capacity contribution of Si and graphite in G–Si electrodes.

Both graphite only electrodes and G–Si composite electrodes have been fabricated with the same manufacturing protocols, as detailed in the Experimental Section. **Figure** **1**a exhibits the cycling performance of these two electrodes. Not surprisingly, addition of 15 weight percent (wt%) of Si into the graphite electrodes nearly triples the initial discharge capacity—918 mAh g^−1^ obtained in the composite electrode, compared to ≈340 mAh g^−1^ in the graphite electrode. However, capacity starts fast degradation after 60 cycles and the G–Si electrode loses most of its reversible charge‐storage capability after 150 cycles. It is noteworthy that the G–Si composite electrode fails to deliver the capacity of 308 mAh g^−1^, which should be achieved by the graphite component alone. This indicates that Si component in the graphite‐based electrode not only loses its own capacity, but also inflict a negative effect on the electrochemical performance of the graphite component. On the contrary, without the addition of Si particles, graphite anodes show remarkably stable reversible capacity for hundreds of cycles.^34^, ^35^

Both graphite and Si components in the G–Si composite electrode can react with Li^+^, as indicated in the differential capacity (d*Q*/d*V*) plots (Figure S1, Supporting Information). During the lithiation process, the cathodic potential peaks corresponding to lithiation of the graphite component significantly overlap with those of the Si component, making the differentiation difficult. In contrast, as shown in Figure 1b, the anodic potential peaks for the graphite are between 0.09 and 0.25 V, whereas the broad peak in a range of 0.25–0.6 V is primarily attributed to the delithiation of Si particles. In Figure S2 (Supporting Information), galvanostatic intermittent titration technique profiles also provide the thermodynamically stable potential ranges for the delithiation process of the Si‐only electrode, graphite‐only electrode, and G–Si electrodes in the first cycle and third cycle. Si and graphite are observed to barely share the potential ranges during delithiation, which strongly supports our aforementioned d*Q*/d*V* data. Therefore, based on the potential separation during the delithiation process, we could distinguish the electrochemical reactivity of each component in the G–Si composite electrodes. The capacity contribution of these two components has also been calculated by integrating d*Q*/d*V* plots with the corresponding voltage range to analyze the effect of Si addition on the electrochemical behavior of the graphite electrode.

Figure 1c displays the total charge capacity of the G–Si composite electrode and the contribution of delithiation capacity from each component. Remarkably, the capacity from the graphite component degrades simultaneously together with Si component. Given the stable electrochemical performance of the graphite anode, this indicates that the Si causes the whole composite electrode to fail. Figure S3 (Supporting Information) plots d*Q*/d*V* from selected cycles and highlights the decrease in the peak area, which is a characteristic signature of electrode activity. It further confirms that the degradation of the Si component finally results in the loss of the electrochemical activity of the whole composite electrode.

### 2.2 Microstructural Study on the Electrochemically Failed G–Si Electrodes

**Figure 2:**
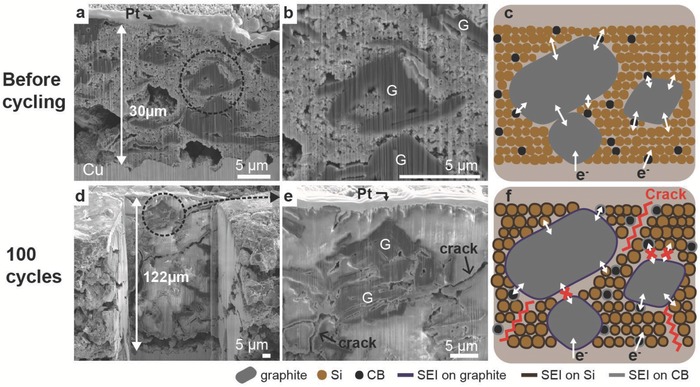
Microstructural investigation on G–Si electrodes before and after cycling. a,b) Cross‐sectional SEM images of G–Si electrode before cycling. c) Schematic of electron pathway in G–Si electrode before cycling. d,e) Cross‐sectional SEM images of G–Si electrode after 100 cycles. f) Schematic of electron pathway in G–Si electrode after cycles.

Focused ion beam (FIB) and scanning electron microscopy (SEM) have been used here to investigate the change in electrode microstructure after 100 cycles. **Figure** **2**a,b shows the cross‐sectional images of the pristine G–Si electrode before cycling. Distribution of both Si nanoparticles and graphite microparticles is highly uniform within the electrode, which has a thickness of 30 µm. The high‐magnification image in Figure 2b further reveals the locational relationships between the graphite and Si in the pristine electrodes. Combining the energy‐dispersive X‐ray spectroscopy (EDS) mapping images (Figure S4, Supporting Information), we confirmed that the physical contacts between graphite, Si, and carbon black (CB) components are well established, as schematically described in Figure 2c, which ensures electronic pathways through the electrode as well as the full utilization of capacities from both components.

However, the internal electrode structure evolves during the electrochemical lithiation and delithiation. After 100 repeated times of lithiation and delithiation, the thickness of the G–Si electrode increases strikingly from 30 to 122 µm, as shown in Figure 2d. The reasons for this large expansion of the G–Si electrode are threefold: (1) Si's volume expands up to ≈300% during the lithiation^2^, ^22^; (2) The volume change occurring during cycling leads to a continuous SEI formation, which consumes the lithium inventory and electrolyte^36^, ^37^; and (3) The colossal volume changes during lithiation/delithiation initiate cracks, which eventual destroys the electrode structure.^36^, ^37^, ^38^ Considering that the graphite electrodes without Si expand less than 10% during lithiation,^39^ the Si particles are seen to contribute significantly to such harmful structure evolution in the G–Si composite electrode. Figure 2e shows the detailed microstructure of the electrode after 100 cycles. Interfaces among solid particles are not as clear as those of the pristine electrodes due to SEI formation on the surface of the particles. Observed cracks generally propagate between solid particles rather than through individual particles. This observation is consistent with previous studies on crack formation in Si anode materials.^40^, ^41^, ^42^ These cracks do not directly cause mechanical failure in the Si nanoparticles themselves, but they do isolate the active materials from connecting to the electrical pathway. Figure 2f schematically displays the microstructure of the G–Si composite electrodes after the electrochemical cycling. Cracks that impede the electrical pathways have been observed in the electrode. Due to the morphology and volumetric changes, a thick SEI layer is expected. Although the SEI layers on the graphite‐only anodes enable stabilization of the structure without exfoliation, the SEI layers are very electronically insulating. Regarding the Si electrode, the thick SEI layer can block the interparticle electron transport between Si–Si and graphite–Si, finally degrading the cycling performance.^35^, ^43^, ^44^, ^45^ It is a little surprising to discover that the electrochemical cycling properties of the composite electrode are dictated by one individual component therein. **Table** **1** summarizes the capacity, weight, volume, and surface‐area percentage for the Si nanoparticles, graphite, and carbon additive. Although accounting for only 15 wt% of the electrode, the Si nanoparticles contribute upto around 86% of the total electrode surface area. In contrast, the graphite, which is the majority (73 wt%) of the mass of the electrode, only accounts for around 1.5% of the total electrode surface area. Given that electrochemical reactions occur at surfaces, the surfaces of the Si nanoparticles provide the major portion of available reaction sites, which explains the overwhelming influence of Si on the electrochemical properties of the G–Si anode. Therefore, surface modification of Si nanoparticles would be an effective approach to improve the electrochemical performance of the G–Si composite electrode.

**Table 1:** The specifications of graphite–Si composite electrode. Particles size of the graphite, Si, and CB are assumed to be 15 µm, 50 nm, and 50 nm, respectively. The values are based on SEM observation and used to calculate the surface area of each component

Component	wt [%]	Capacity	Volume [%]	Surface area [%]
Graphite	73 wt%	308 mAh g^−1^	81.49%	1.45%
Si	15 wt%	610 mAh g^−1^	16.25%	86.51%
CB	2 wt%	N/A	2.26%	12.04%
PAA	10 wt%	N/A	N/A	N/A

### 2.3 Surface Modification of G–Si Electrodes with Alucone‐MLD Coating

**Figure 3:**
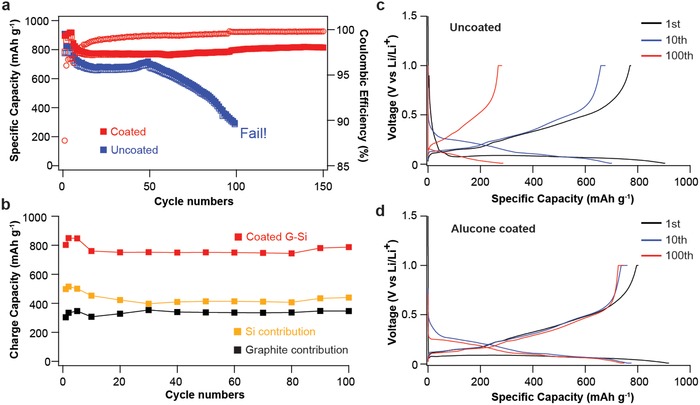
Electrochemical performances of G–Si electrodes with and without alucone‐MLD coating. a) Cycling performances of G–Si electrodes without (blue) and with (red) alucone coating. b) Charge‐capacity contribution of Si and graphite in alucone‐coated G–Si electrodes. c) Voltage profiles of uncoated G–Si electrode. d) Voltage profiles of alucone‐coated G–Si electrode.

To mitigate failures of Si surface in the G–Si composite caused by microstructural expansion/crack formation and excessive SEI formation, an alucone‐based surface modification was applied by MLD on laminated G–Si electrodes. The synthesis of the alucone coating has been detailed in the Experimental Section. As recently reported by our group, the alucone‐MLD coating has shown significant positive impact on cycling performances because of its mechanical resilience and suppression of electrode expansion during cycling.^27^, ^28^, ^29^, ^30^ Chemical evolution of SEI on the coated electrodes, which has not been previous investigated, is elaborated in the Figures 6 and 7. Galvanostatic charging and discharging were performed in a half‐cell configuration with Li metal as the counter electrode. The electrochemical cycling data from both the uncoated and coated electrodes are compared in **Figure** **3**a. Cells were cycled at a rate of 0.04 C (36.72 mA g^−1^) for the first five cycles, then 0.1 C (91.8 mA g^−1^) for the subsequent cycles between 10 mV to 1 V. As shown in Figure 3a, the G–Si electrode (plotted in blue) shows capacity degradation after 50 discharge/charge cycles and loses most of capacity within 100 cycles. In strong contrast, the alucone‐coated G–Si electrode (plotted in red) demonstrates significant improvement in the cycling stability, achieving a reversible capacity of 814 mAh g^−1^ with the CE of 99.80% for more than 100 cycles. The improved cycling performance can be better explained with Figure 3b, which shows the capacity contributed by both Si and graphite components. It suggests that the stable capacity retention of alucone‐coated G–Si electrodes is enabled by highly reversible capacity contributions from both the Si and graphite components. Capacity retention of these two components is 100.1% for graphite and 85.46% for Si at the 100th cycle. The capacity retention of the Si component is lower compared to that of the graphite component, but it is significantly improved over the capacity retention of an uncoated graphite–Si composite or Si‐only electrodes. Figure S5 (Supporting Information) shows the differential capacity plots for the alucone‐coated G–Si electrodes at different cycling numbers.

Figure 3c,d present the galvanostatic lithiation and delithiation voltage profiles obtained from the uncoated and coated G–Si electrodes at selected cycle numbers. Lithiation occurs at around 0.1 V for both the graphite and Si components for the first cycle. Figure S6 (Supporting Information) compares the initial voltage profiles of the uncoated and coated G–Si electrodes. After the first cycle, the Si nanoparticles exhibit a sloping lithiation voltage profile due to the amorphization of crystalline Si. Si capacity of the uncoated electrode is significantly reduced from 918 mAh g^−1^ at the first cycle down to ≈270 mAh g^−1^ at the 100th cycle, with an increase in overpotential with increased cycle number. In dramatic contrast, the voltage profiles of the alucone‐coated G–Si electrode (Figure 3d) remain unchanged during continuous cycling, indicating excellent electrochemical reversibility with much higher capacity and lower voltage hysteresis than those of the uncoated G–Si electrodes.

**Figure 4:**
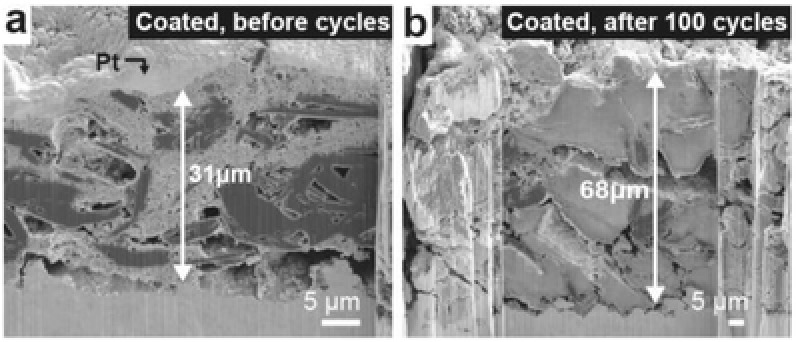
Microstructural investigation on alucone‐coated G–Si electrodes before and after cycling. a) Cross‐sectional SEM images of alucone‐coated G–Si electrode before cycling. b) Cross‐sectional SEM images of alucone‐coated G–Si electrode after 100 cycles.

The impact of the coating on the electrode microstructure is observed using the FIB‐SEM approach. The electrode microstructure before and after 100 cycles, as shown in **Figure** **4**a,b, has been well preserved. The uncoated G–Si electrode expands its thickness to 122 µm (Figure 2d), whereas the alucone‐coated G–Si electrode only expands its thickness to 68 µm. The results achieved in Figure 4 are in agreement with our previous microstructural analysis that an alucone coating can successfully maintain electrode structure and suppress increases in net electrode volume.^22^, ^24^ And Figure S7 (Supporting Information) presents further microstructural observations on surface and cross‐section of both uncoated and alucone‐coated G–Si electrodes after 25 cycles. In Figure S8 (Supporting Information), EDS mapping results of Figure 4 further confirm the well‐preserved structure comprising uniformly distributed Si nanoparticles on the graphite microparticles. Alucone coating on the G–Si anode effectively improves the mechanical resiliency and elasticity of the electrode; therefore, it preserves the physical contacts among solid particles and maintains the electrical pathway within the G–Si electrodes during electrochemical cycling.

### 2.4 EIS Analysis on the Electrochemically Cycled G–Si Electrodes

**Figure 5:**
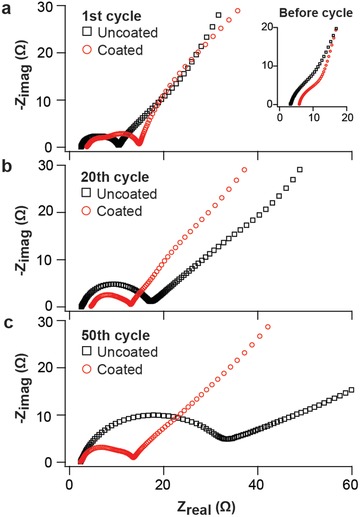
The EIS measurements on uncoated (black) and alucone‐coated (red) G–Si electrodes at a) 1st cycle, b) 20th cycle, and c) 50th cycle.

We used electrochemical impedance spectroscopy (EIS) to evaluate the conductivity of the electrodes and the impedance evolution with increased cycle numbers. **Figure** **5** compares EIS for the G–Si electrodes with and without the alucone coating, indicating the conductivity/impedance evolution during electrochemical cycling. All of the EIS data were collected in symmetric cells composed of uncoated G–Si electrodes (black) and alucone‐MLD‐coated G–Si electrodes (red). Each cell was cycled against Li counter electrodes, then disassembled to build symmetric cells (G–Si/G–Si configuration). The impedance data were obtained at different cycle numbers: the uncycled state (inset of Figure 5a), first cycle (Figure 5a), 20th cycle (Figure 5b), and 50th cycle (Figure 5c). In the Nyquist plots, the high‐frequency intercept relates to the Ohmic resistance (*R*_s_) and includes the contributions from the electronic conductivity of the electrodes and ionic conductivity of the electrolyte. The frequency region between 100 kHz and 10 Hz is generally assigned to the interface resistance comprising SEI resistance (*R*_SEI_) for high frequency, and charge transfer resistance (*R*_ct_) for medium frequency.^16^, ^46^, ^47^ The low‐frequency range between 10 and 1 Hz is generally a feature of Li‐ion diffusion inside the electrodes. And in the observed Nyquist plots, the diameter of semicircles originates from the interface resistances, which are the sum of *R*_CT_ and *R*_SEI_. In the inset of Figure 5a, initial *R*_s_ is observed to be slightly higher for the coated G–Si electrodes compared to that of uncoated G–Si, which could be attributed to the additional alucone layer on the electrode's surface. Also, in the first cycle, a larger *R*_SEI_ is observed in the alucone‐coated G–Si cell, consistent with the observation of lower initial capacity in the alucone‐coated G–Si cell data in Figure 3. Surface resistances after the 20th and 50th cycles are significantly higher for uncoated G–Si electrodes compared to the alucone‐coated G–Si electrodes. The notable observation is that the interface resistances for alucone‐coated G–Si electrodes remain relatively unchanged whereas uncoated G–Si electrodes build considerable resistances after 50 cycles. In this study, EIS was measured at the same state‐of‐charge (SOC) level for all cases. Therefore, variations in the interface resistances of G–Si electrodes are not caused by lithiation/delithiation status of G–Si electrodes, but rather, principally by the different interfacial film formation on the surface. The fitting results of Figure 5 and the corresponding equivalent circuit are provided in Figure S9 in the Supporting Information.

### 2.5 Interfacial Analysis of G–Si Electrodes Using XPS

**Figure 6:**
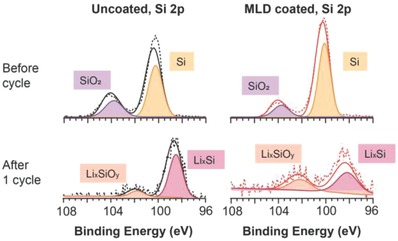
Si 2p spectra of uncoated and alucone‐MLD‐coated G–Si electrodes before and after first cycle.

To elucidate the precise compositional evolution of the surfaces of the G–Si electrodes including SEIs, X‐ray photoelectron spectroscopy (XPS) was used to verify the chemical bonding of the surface species after the electrodes are electrochemically cycled. In **Figure** **6**, the Si 2p spectra have been collected both before and after the initial cycle. Both electrodes show a strong Si (100.1 eV) and SiO_2_ peak (103.7 eV) with relatively low intensity.^48^ It is known that native oxide on the Si surface oxide layer is removed during the MLD process.^30^ Relatively lower SiO_2_ intensity for the MLD‐coated electrode (17.03% for SiO_2_ and 82.97% for Si) compared to the uncoated electrode (29.76% for SiO_2_ and 70.24% for Si) is observed before cycle. After the first cycle, both Si and SiO_2_ peaks disappear and are replaced by peaks of Li*_x_*Si and Li*_x_*SiO*_y_*, which are caused by irreversible Li loss in Si.^48^

**Figure 7:**
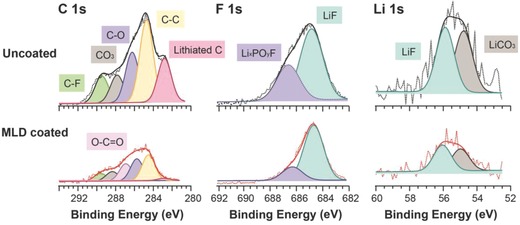
XPS spectra of uncoated and alucone‐MLD‐coated G–Si electrodes after 100 cycles.

**Figure** **7** shows XPS spectra of C 1s, F 1s, and Li 1s of coated and uncoated G–Si electrodes. Cells are cycled 100 times to investigate the evolution of SEI with respect to the existence of the alucone‐MLD layer. The G–Si electrodes with 100 cycles reveal notable electrochemical performance differences depending on whether G–Si electrodes are alucone‐MLD‐coated or not. Infrared (IR) and XPS analyses on SEI components on the graphite and Si surfaces have been studied extensively, and the chemical components of lithium ethylene dicarbonate (LEDC), LiCO_3_, and LiF are known as main components of the SEI.^49^, ^50^, ^51^, ^52^ These SEI components are consistently observed in our XPS analysis in Figure 7. However, certain different characteristics between uncoated and alucone‐coated G–Si electrodes are observed. The lithiated carbon peak (282.8 eV) is shown at both electrodes, but with much higher intensity for the uncoated G–Si electrode. Detailed atomic concentrations can be found in Figure S8 in the Supporting Information. The lithiated carbon originates from the graphite particles that are lithiated during continuous electrochemical cycles. Given that the electrodes are disassembled at delithiated status for the XPS measurements, those peaks indicate the amount of electrochemically dead graphite particles with trapped Li in the composite electrodes. The amount of dead graphite is much larger for the uncoated G–Si anode compared to the alucone‐coated G–Si anode, as discussed in Figure 2. Note that C 1s spectra in MLD‐coated G–Si electrode show negligible peak intensity of the lithiated carbon. In addition, LiF is more prominent compared to the Li*_x_*PO*_y_*F species in the alucone‐MLD‐coated electrode, indicating that the electrolyte has undergone less reductive decompositions and resulting in a reduced appearance of the Li*_x_*PO*_y_*F species.^53^

## 3 Conclusion

Addition of 15 wt% of Si into the graphite‐based electrodes results in the rapid failure of the entire graphite–Si composite electrode. Using differential voltage plots and integrations, we were able to quantitatively distinguish the capacity contributions from the two active materials—graphite and Si. The established methodology reveals that graphite loses its intrinsic capacity after going through charge/discharge cycles in the graphite–Si composite electrode. The FIB‐SEM observation suggests that the graphite particles become isolated from the electrical network due to the deteriorating microstructure of the electrodes caused by Si particle volume expansion, cracks, and excessive SEI formation at the interface. XPS analysis after delithiation confirms the existence of lithiated graphite, which is assumed to be isolated graphite in the composite electrode. An alucone‐MLD coating was applied on the composite electrodes as a strategy to improve the electrochemical and microstructural instabilities of graphite–Si composite electrodes. After the surface modification with alucone‐MLD, electrochemical performance of the graphite–Si composite electrode is greatly improved, with only 38.8% volume expansion compared to uncoated electrodes. The use of an alucone coating on graphite–Si composite electrodes will be further studied and developed because it mitigates Si volume expansion during cycling. The alucone coating thus helps to maintain a higher G–Si composite‐anode energy‐storage capability than graphite‐only anodes. The analytical method established here provides a general tool to quantitatively describe the electrochemical behavior of individual active species in a multicomponent composite, which could be applied on numerous systems in the next generation battery chemistries that face similar challenges. The alucone mitigation strategy is expected to be of high interest to mitigate such challenges.

## 4 Experimental Section

*Microstructure Analysis*: A FIB (FEI, NOVA 200 dual‐beam system) was used for cross‐sectional SEM observation and EDS line scanning. A Ga‐ion source was used for FIB sectioning.

*Preparation of the Electrodes and Coin Cells*: Standard CR2032 coin cells with Li metal foil as counter electrodes were prepared for these experiments. The anode mixture was composed of graphite, Si, carbon black, and poly acrylic acid (PAA) binder with a wt% ratio of 73%:15%:2%:10% and mixed with a 1‐methyl‐2‐pyrrolidinone solution. The mixture was coated on Cu foil and then dried under air. Before assembling the cells, punched electrodes (diameter of 1.4 cm) were dried overnight (100 °C) in a vacuum oven. 1.2 m LiPF_6_ in ethylene carbonate:ethyl methyl carbonate (3:7 by weight) with 10 wt% fluoroethylene carbonate was used as the electrolyte. Cells were assembled in an Ar‐filled glove box and tested at room temperature.

*Electrochemical Measurements*: Constant current was applied during discharge and charge between the voltage range of 0.01–1.0 V. Cycling performances were carried out using a Maccor battery test station. Electrochemical impedance spectroscopy was performed using a Biologic VMP3. AC impedance measurements were recorded using a signal with amplitude of 5 mV and a frequency from 1 MHz to 5 mHz. EIS tests were always conducted at a SOC of 0.2 V versus Li/Li^+^.

*Alucone Coating on the Electrodes*: Alucone films were grown directly on the G–Si electrodes using the trimethylaluminum (TMA)/glycerol MLD process. Static precursor exposures were used to give the precursors sufficient time to diffuse into the high‐aspect‐ratio electrode structures. The electrodes were heated to 150 °C and dried under a low‐pressure nitrogen flow in a hot‐wall MLD reactor chamber. The glycerol was heated to 100–120 °C. The alucone reaction sequence was the following: i) dose TMA; ii) hold TMA pressure static for 60 s; iii) pump down; iv) repeat i), ii), and iii) until the mass spectrometer indicates a saturated substrate surface; v) pump down and nitrogen purge; vi)–x) repeat the above procedure with glycerol. This sequence constitutes one cycle of the alucone‐MLD process. The electrodes were coated with ten cycles of alucone. The alucone reaction sequence was the following: i) dose TMA until the mass spectrometer indicates saturation of the TMA surface reaction; ii) purge with nitrogen; iii) dose glycerol until the mass spectrometer indicates saturation of the TMA surface reaction; iv) purge with nitrogen. This sequence constitutes one cycle of the alucone‐MLD process. The electrode was coated with ten cycles of alucone.

*XPS Measurements*: XPS was performed on a PHI Versaprobe 3 instrument with a monochromated Al kα X‐ray source using a spot size of 100 µm and a spot energy of 25 W. Samples were loaded into a sealed container in an argon‐filled glovebox with moisture content of less than 1 ppm H_2_O and an oxygen content of 1–3 ppm. Samples were then transferred directly to the XPS sample chamber such that they were not exposed to the outside environment. Elemental scans were conducted with a pass energy of 55 and a 0.04 eV step size with constant sample and beam neutralization during the count collection process. Raw data were analyzed and deconvoluted using PHI MultiPak software, taking the C–C characteristic peak at 284.8 eV.

## Conflict of Interest

The authors declare no conflict of interest.

## Supporting information

Supplementary
