# Supplementary material for: Interfacially Induced Cascading Failure in Graphite‐Silicon Composite Anodes
Source: Adv Sci (Weinh). 2018 Dec 14;6(3):1801007. doi: 10.1002/advs.201801007 (PMC6364491; doi:10.1002/advs.201801007)
Supplement: Supplementary file 1 — Supplementary [file ADVS-6-1801007-s001.pdf]

Copyright WILEY-VCH Verlag GmbH & Co. KGaA, 69469 Weinheim, Germany, 2018.

Supporting Information

for *Adv. Sci.,* DOI: 10.1002/advs.201801007

Interfacially Induced Cascading Failure in Graphite-Silicon Composite Anodes

*Seoung-Bum Son, Lei Cao, Taeho Yoon, Arthur Cresce, Simon E. Hafner, Jun Liu, Markus Groner, Kang Xu, and Chunmei Ban**

Supporting Information

Interfacially Induced Cascading Failure in Graphite-Silicon Composite Anodes

Seoung-Bum Son,[a] Lei Cao,[a] Taeho Yoon,[a] Arthur Cresce,[b] Simon Hafner,[a] Jun Liu,[a] Markus Groner,[c] Kang Xu,[b] Chunmei Ban[a,*]

[a] National Renewable Energy Laboratory, 15013 Denver West Parkway, Golden, CO 80401, USA

[b] Electrochemistry Branch, Sensor and Electron Devices Directorate, U.S. Army Research Laboratory, Adelphi, MD 20783-1197, USA

[c] ALD NanoSolutions, 580 Burbank Street, Unit 100, Broomfield, CO 80020, USA

* Corresponding author: Dr. Chunmei Ban

E-mail: chunmei.ban@nrel.gov, Tel.: 303-384-6504, Fax: 303-384-6490

Supplementary Figure 1

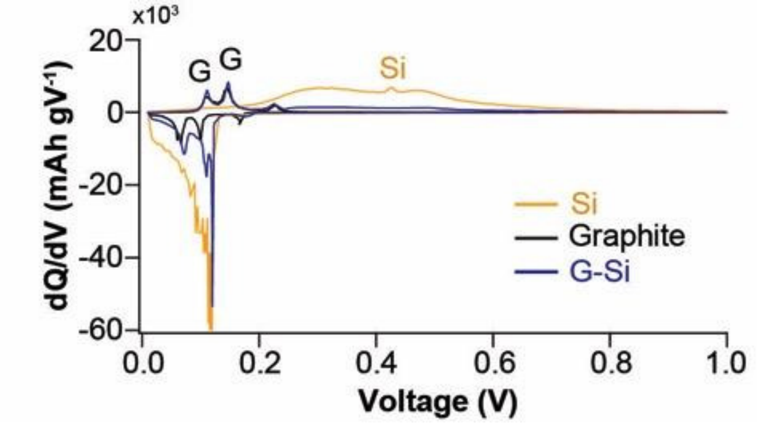

Supplementary Figure S1. Differential plots for first cycle of Si, graphite, and G-Si composite electrodes. Both Si and graphite use a potential range of 0.1 V to 10 mV for its lithiation whereas delithiation occurs mainly between 0.09 V and 0. 25 V for the graphite and 0.2 V to 0.65 V for the Si. This observation leads us to use oxidation part to distinguish the capacity contribution of each component—Si and graphite—in the G-Si composite electrode.

Supplementary Figure 2

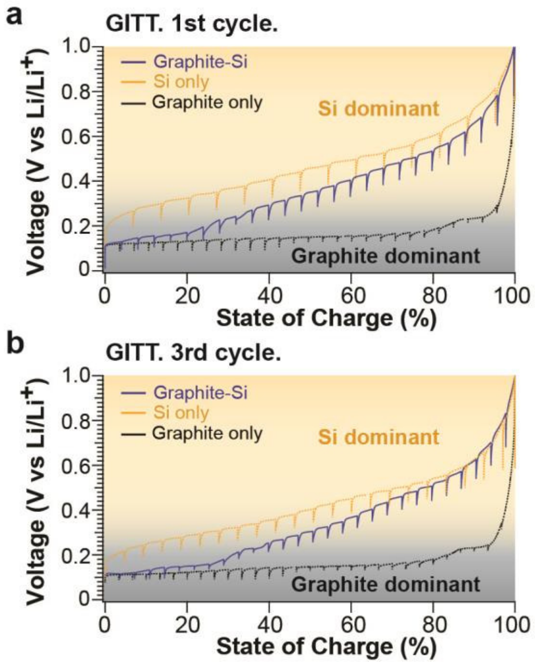

Supplementary Figure S2. GITT curves of Si, graphite, and G-Si electrodes. Delithiation regime is only focused to show thermodynamically stable potential range of those electrodes. (a) GITT of first cycle. (b) GITT of third cycle. The electrodes are pre-cycled at a rate of 0.05 C for 2 cycles, then GITT was performed.

GITT was performed by using a rate of 0.05 C and stopping every 3% of SOC followed by a 4 h relaxation within the voltage range of 10 mV to 1 V.

Supplementary Figure 3

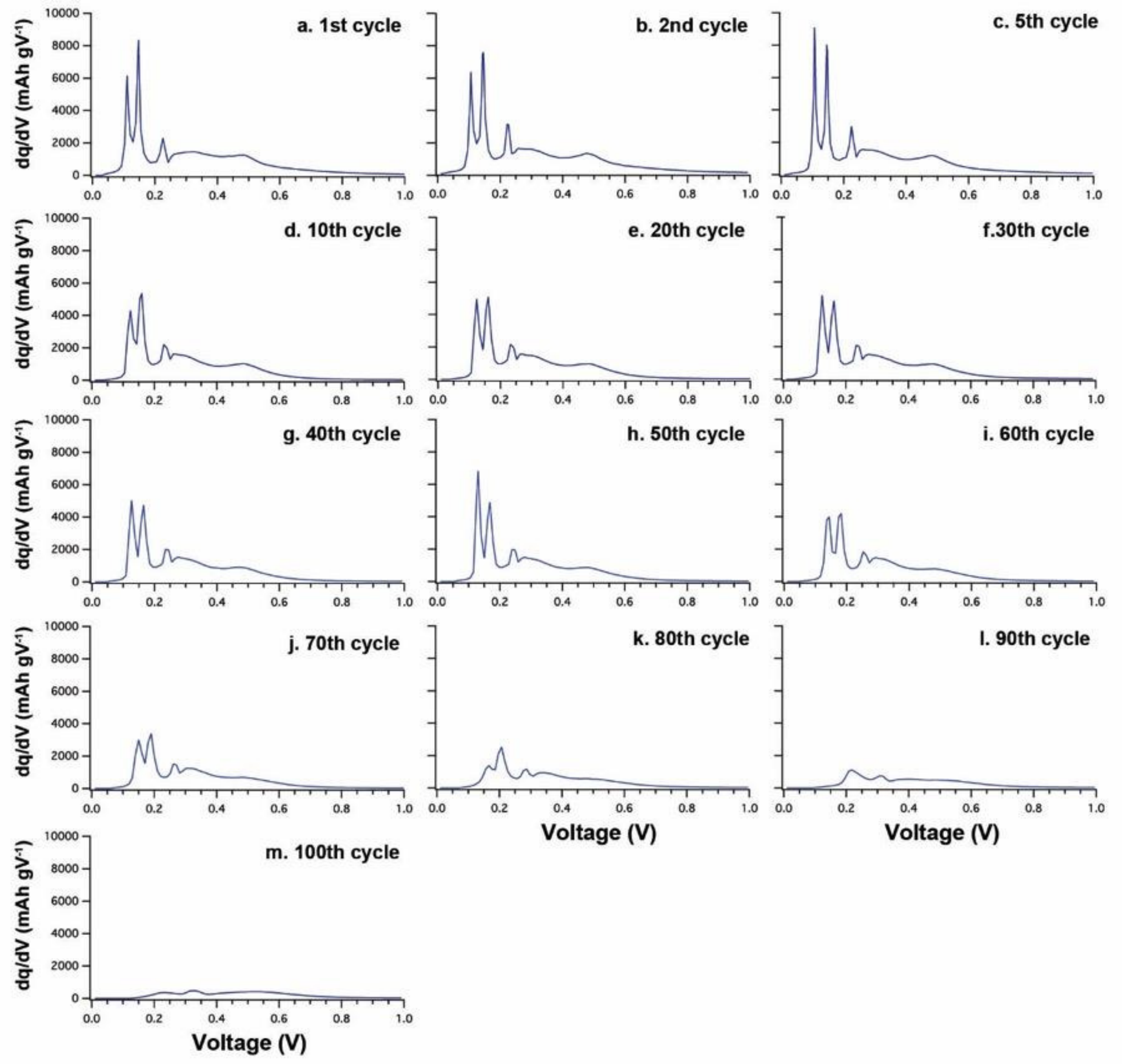

Supplementary Figure S3. Differential capacity plots of G-Si composite electrode. (a) 1st cycle, (b) 2nd cycle, (c) 5th cycle, (d) 10th cycle, (e) 20th cycle, (f) 30th cycle, (g) 40th cycle, (h) 50th cycle, (i) 60th cycle, (j) 70th cycle, (k) 80th cycle, (l) 90th cycle, and (m) 100th cycle.

Supplementary Figure 4

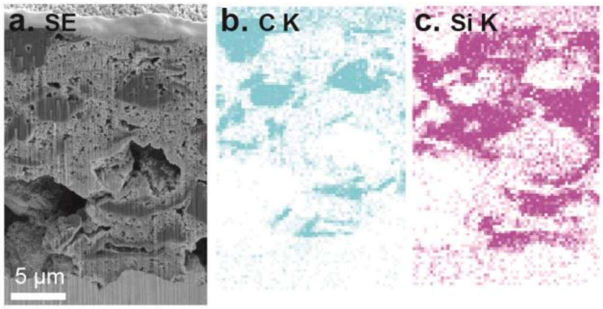

Supplementary Figure S4. EDS mapping images of G-Si electrode. (a) Secondary electron image. (b) Carbon mapping image. Carbon represents graphite and carbon black in the electrode. (c) Si mapping image.

Supplementary Figure 5

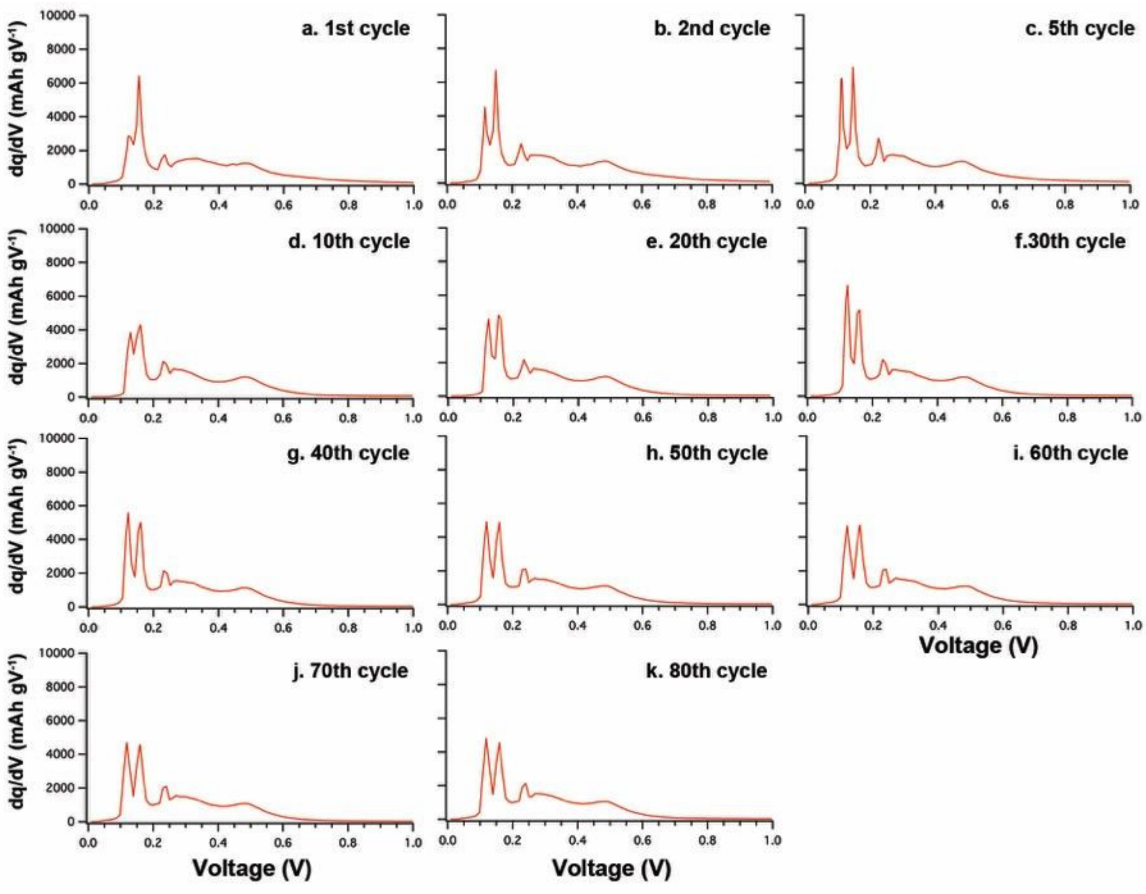

Supplementary Figure 5. Differential capacity plots of alucone-MLD-coated G-Si composite electrode. (a) 1st cycle, (b) 2nd cycle, (c) 5th cycle, (d) 10th cycle, (e) 20th cycle, (f) 30th cycle, (g) 40th cycle, (h) 50th cycle, (i) 60th cycle, (j) 70th cycle, (k) 80th cycle, (l) 90th cycle, and (m) 100th cycle.

Supplementary Figure 6

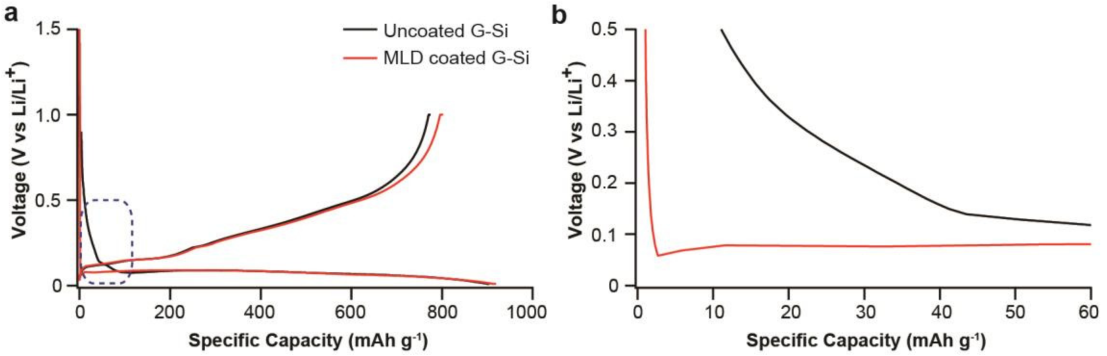

Supplementary Figure S6. Initial voltage profiles of the uncoated (black) and coated (red) G-Si electrodes. (a) shows the whole region of initial voltage profiles and (b) enlarge the initial lithiation region indicated with dotted line in (a).

We observe that the discharge plateau of the alucone coated Si anode occurs at a lower voltage than that of the uncoated Si anode. It has been reported that a compressive stress applied on Si particles can lower the discharge voltage plateau.[1] In this case, alucone-MLD coating confines the free volume expansion of the Si particles during Li insertion and manifested as a lower discharge voltage plateau during initial lithiation.

Supplementary Figure 7

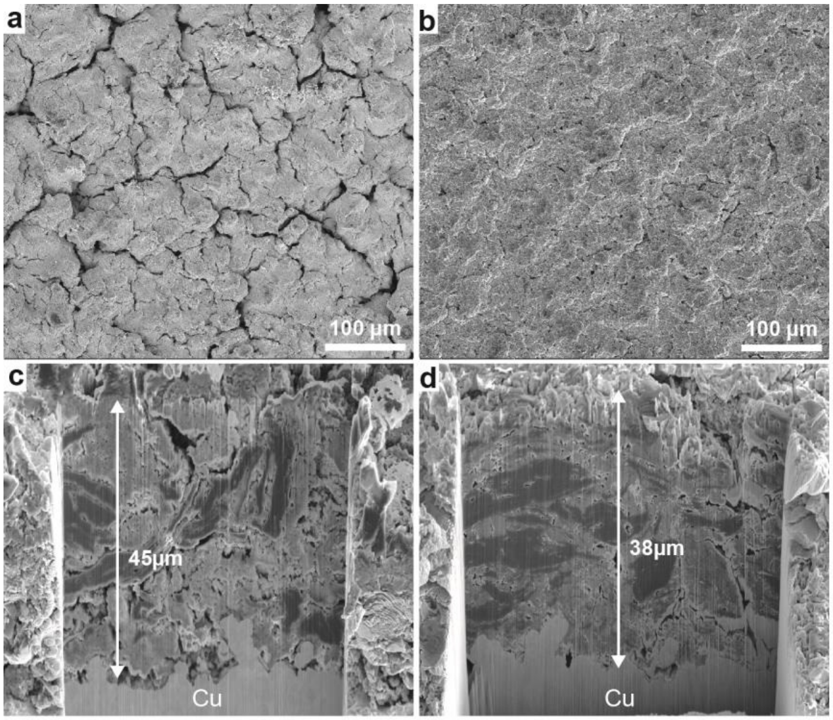

Supplementary Figure S7. SEM images of (a) the uncoated electrode after 25 electrochemical cycling, (b) the coated electrode after 25 electrochemical cycling. The cross-section images of (c) the uncoated electrode after 25 electrochemical cycling, and (d) the coated electrodes after 25 electrochemical cycling. Both the uncoated and coated electrodes have been soaked in DMC for overnight before characterization.

FigureS7 compares the surface morphology and the cross-section images of both uncoated and alucone-coated electrodes after 25 cycles. A large number of cracks have been observed for the uncoated electrode, after removing the SEI, as indicated in Figure S7-a. However, the original morphology of the electrode has been maintained for the coated electrode, as seen in Figure S7-b. A number of cracks observed on the uncoated electrode should be due to the repeated volume changes during cycling and manifested as

increased electrode thickness to 45 μm as shown in Figure S7-c. Notice that coated electrode only increased to 38 μm as presented in Figure S7-d.

Supplementary Figure 8

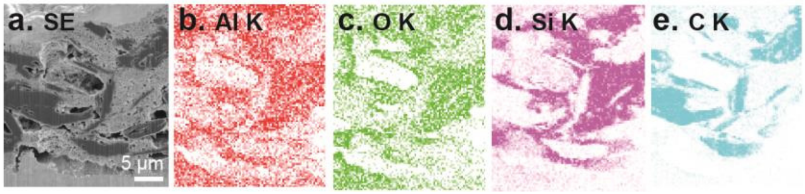

Supplementary Figure S8. EDS mapping images of alucone-coated G-Si electrode. (a) Secondary electron image. (b) Al mapping image. (c) O mapping image. Al and O are the main component for the alucone. (d) Si mapping image. (e) Carbon mapping image.

Supplementary Figure 9

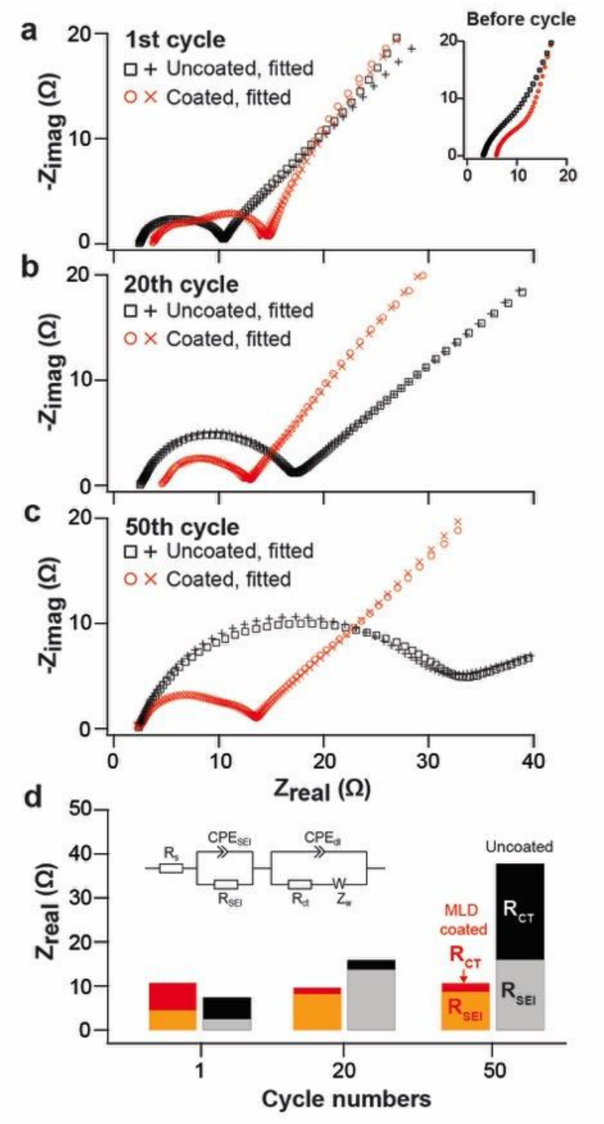

Supplementary Figure S9. The EIS measurements and fitting results on uncoated (black) and alucone-coated (red) G-Si electrodes at (a) 1st cycle, (b) 20th cycles, and (c) 50th cycles. (d) Distribution of R_{SEI} and R_{ct} achieved by calculations using the equivalent circuit in the inset of Figure S9a.

	Assignment	Binding energy (eV)		Atomic concentration (%)	
		Uncoated	Coated	Uncoated	Coated
Li 1s	$LiCO_3$	54.79	54.98	48.74	45.82
	LiF	55.88	56.05	51.26	54.18
C 1s	Lithiated carbon	282.8	282.9	18.86	2.87
	C-C	284.7	284.5	35.62	30.65
	C-O	286.2	285.8	21.78	26.02
	O-C=O		286.9		20.32
	CO_3	287.8	288.3	11.98	11.11
	C-F	289.5	289.6	11.75	9.03
F 1s	LiF	684.9	684.7	66.61	80.33
	Li_xPO_yF	686.6	686.3	33.39	19.67

Supplementary Table 1.

Supplementary Table 1. Atomic concentrations for the chemical components of XPS analysis shown in Figure 7.

References

[1] V. A. Sethuraman, V. Srinivasan, A. F. Bower, P. R. Guduru, *J. Electrochem. Soc.* **2010**, *157*, A1253.
